# Supplementary material for: Modelling Skylarks (Alauda arvensis) to Predict Impacts of Changes in Land Management and Policy: Development and Testing of an Agent-Based Model
Source: PLoS One. 2013 Jun 6;8(6):e65803. doi: 10.1371/journal.pone.0065803 (PMC3675089; doi:10.1371/journal.pone.0065803)
Supplement: Supporting Information S4 — The skylark ODdox as a zipped archive. (ZIP) [file pone.0065803.s004.zip › Skylark_ODdox/functions_0x7e.html]

ALMaSS Skylark ODdox: Class Members


ALMaSS Skylark ODdox
 2.0


- Main Page
- Related Pages
- Classes
- Files

- Class List
- Class Index
- Class Hierarchy
- Class Members

- All
- Functions
- Variables
- Typedefs

- a
- b
- c
- d
- e
- f
- g
- h
- i
- j
- k
- l
- m
- n
- o
- p
- r
- s
- t
- u
- v
- w
- x
- y
- z
- ~

Here is a list of all class members with links to the classes they belong to:

### - ~ -

- ~BaitLocation()
  : BaitLocation
- ~BinaryMapBase()
  : BinaryMapBase
- ~CfgBase()
  : CfgBase
- ~Configurator()
  : Configurator
- ~Crop()
  : Crop
- ~CropData()
  : CropData
- ~CropRotation()
  : CropRotation
- ~Farm()
  : Farm
- ~FarmManager()
  : FarmManager
- ~GreenElement()
  : GreenElement
- ~IDMap()
  : IDMap
- ~Landscape()
  : Landscape
- ~LE()
  : LE
- ~MovementMap()
  : MovementMap
- ~MovementMap16()
  : MovementMap16
- ~Pesticide()
  : Pesticide
- ~PolygonDataVector()
  : PolygonDataVector
- ~Population\_Manager()
  : Population\_Manager
- ~PositionMap()
  : PositionMap
- ~probe\_data()
  : probe\_data
- ~RasterMap()
  : RasterMap
- ~RodenticideManager()
  : RodenticideManager
- ~ScalablePositionMap()
  : ScalablePositionMap
- ~SimplePositionMap()
  : SimplePositionMap
- ~SkTerritories()
  : SkTerritories
- ~Skylark\_Adult()
  : Skylark\_Adult
- ~Skylark\_Male()
  : Skylark\_Male
- ~Skylark\_Population\_Manager()
  : Skylark\_Population\_Manager
- ~Skylark\_struct()
  : Skylark\_struct
- ~TALMaSSObject()
  : TALMaSSObject
- ~VegElement()
  : VegElement
- ~Weather()
  : Weather


- Generated on Thu Jan 10 2013 13:15:39 for ALMaSS Skylark ODdox by
   1.8.1.1
